# Atypical low-frequency and high-frequency neural entrainment to rhythmic audiovisual speech in adults with dyslexia

**DOI:** 10.1162/IMAG.a.1329

**Published:** 2026-08-05

**Authors:** Mahmoud Keshavarzi, Brian C.J. Moore, Usha Goswami

**Affiliations:** Centre for Neuroscience in Education, Department of Psychology, University of Cambridge, Cambridge, United Kingdom; Cambridge Hearing Group, Department of Psychology, University of Cambridge, Cambridge, United Kingdom

**Keywords:** developmental dyslexia, rhythmic audiovisual speech, EEG, neural phase entrainment, low-frequency bands, high-frequency bands

## Abstract

Developmental dyslexia has been linked to atypical neural processing of the temporal dynamics of speech, but there has been disagreement concerning whether faster or slower dynamics are impaired. According to the temporal sampling (TS) theory, dyslexia arises from impaired entrainment of low-frequency neural oscillations—particularly in the delta (1–4 Hz) and theta (4–8 Hz) bands—to the rhythmic modulations of speech. This hypothesis was tested for adults with and without dyslexia using electroencephalography (EEG) and employing a rhythmic audiovisual speech paradigm previously used with dyslexic children. Participants viewed a “talking head” repeating the syllable “ba” at a 2-Hz rate. Measures were neural phase entrainment and band power in the delta, theta, beta (15–25 Hz), and low gamma (25–40 Hz) bands. Phase–amplitude coupling (PAC) and phase–phase coupling (PPC) were also assessed for delta–theta, delta–beta, theta–beta, delta–gamma, and theta–gamma interactions. Both groups exhibited significant delta- and theta-band phase entrainment; however, the two groups differed significantly in the preferred phase for the theta band. While the control group showed consistent beta- and low gamma-band phase entrainment, this was not observed for the dyslexic group. There was significantly greater delta-band power for the dyslexic group across the whole brain and in the right temporal region. These findings indicate that temporal sampling deficits appear to persist into adulthood and show a multi-timescale pattern, with altered low-frequency processing and atypical higher-frequency entrainment. The data also generate developmental hypotheses about possible compensation or reorganisation mechanisms that could be examined in future longitudinal research.

## 1 Introduction

Dyslexia is a developmental disorder characterised by impairments in reading, writing, and spelling, primarily attributed to a core difficulty in phonological processing (Rack, 1994; Ramus, 2004). This “phonological deficit” is characterised by difficulties in recognising, memorising, and manipulating sounds in words, which are essential precursor skills for learning to read (Lyon et al., 2003). Affecting approximately 5–10% of the population (Roitsch & Watson, 2019), dyslexia is not associated with impaired general intelligence, reasoning, and intellectual abilities, but poses substantial challenges in academic environments and daily life (Shaywitz, 1998). A central aspect of the phonological difficulties is poor phonological awareness, defined as the capacity to recognise and manipulate oral units such as syllable stress patterns, syllables, onset-rimes, and phonemes within spoken language (Goswami et al., 2021). These challenges likely arise from atypical neural processing within brain regions critical for language and auditory functions, and may involve neural oscillatory systems (Goswami, 2011; Lehongre et al., 2011; Tallal & Piercy, 1973). The temporal sampling (TS) theory (Goswami, 2011, 2015, 2018) proposes that dyslexia is characterised by deficits in accurately processing slower temporal characteristics of speech sounds, with frequencies below 10 Hz, primarily affecting the delta and theta oscillatory networks. Specifically, TS theory suggests that individuals with dyslexia struggle to perceive and process the precise timing and patterns of amplitude modulation associated with syllabic structures, the parsing of which is related to slower rates of temporal modulation. In contrast, other theories focus on rapid processing deficits in temporal windows corresponding to frequencies in gamma-band oscillatory networks (Lehongre et al., 2011; Tallal, 2004), although atypical responses have also been reported in people with dyslexia at frequencies around 20 Hz, within the beta range (Poelmans et al., 2012). Tallal’s Rapid Auditory Processing (RAP) theory and the “phonemic oversampling” proposed by Lehongre et al. (2011) focus on phoneme-level structures, which are related to faster rates of temporal modulation. However, given the mechanistic hierarchical nesting between slow (theta) and fast (gamma) oscillatory rates in auditory cortex (Gross et al., 2013), it is possible that atypical low-frequency temporal sampling may subsequently result in altered fast-rate activity. Any level of impaired temporal processing is assumed to hinder the development of phonological awareness in infancy and childhood, complicating subsequent learning of the mapping between written letters and their corresponding sounds.

Importantly, these theoretical perspectives raise a broader question regarding the nature of temporal sampling deficits in dyslexia: whether such deficits are specific to slow frequency processing, specific to faster frequency processing, or reflect a more general disruption of hierarchical temporal organisation across multiple frequency bands. To address this gap, the current study adopts a multi-level approach, combining several complementary neural measures. Phase entrainment provides a direct index of the alignment of neural oscillations to rhythmic input and constitutes the core mechanism proposed by the TS framework. Band-power analyses quantify the magnitude of oscillatory activity within each frequency band, offering complementary information about the strength of neural engagement at different temporal scales. Cross-frequency coupling measures (phase–amplitude coupling [PAC] and phase–phase coupling [PPC]) index the coordination between oscillatory processes operating at different temporal scales, reflecting the hierarchical organisation of neural processing. Together, these measures allow us to test whether dyslexia reflects frequency-specific impairments, broadband temporal sampling disruption, or altered coordination across neural timescales.

Neural phase entrainment has been studied intensively in dyslexia, as it is one of the mechanisms that enables the brain to align temporally with external rhythmic stimuli, thus facilitating efficient processing of rhythmic information (Lakatos et al., 2008). In the rhythmic syllable repetition task, children with dyslexia exhibit atypical phase entrainment in both the delta and beta frequency bands (Keshavarzi et al., 2022; Keshavarzi, Mandke, et al., 2024; Power et al., 2013). Prior studies of neural entrainment in adults have utilised non-speech rhythmic stimuli, most typically amplitude-modulated (AM) noise (Lehongre et al., 2011; McAnally & Stein, 1997; Menell et al., 1999; Poelmans et al., 2012). However, they have either not measured oscillations in the delta band (~2 Hz, Poelmans et al., 2012), or have not measured oscillations in both the delta and theta bands (modulations <10 Hz, Lehongre et al., 2011; McAnally & Stein, 1997; Menell et al., 1999). Using AM white noise, McAnally and Stein (1997) reported reduced Auditory Steady State Response (ASSR) power to AM with rates from 20 to 80 Hz for dyslexic adults compared with controls. Menell et al. (1999) measured ASSR power for AM rates of 10, 20, 40, 80, and 160 Hz and reported that the ASSR power was lower for dyslexic readers than for controls for all AM rates. However, both these studies used only a single electrode. Lehongre et al. (2011) used a white noise stimulus with a range of AM rates (10–80 Hz), which they argued broadly covered the phonemic sampling domain. Using magnetoencephalography (MEG), they demonstrated lower left-lateralised low-gamma (~30 Hz) responses for dyslexic adults than for typically reading control adults, which was interpreted to show impaired entrainment to phonemic-rate modulations in left auditory cortex. Intriguingly, they also observed enhanced cortical entrainment at rates >40 Hz for the dyslexic adults, which they reported was linked to impaired phonological memory. Using EEG, Poelmans et al. (2012) administered speech-weighted noise AM at 4, 20, and 80 Hz to measure ASSRs in dyslexic adults. They employed a limited EEG montage (recordings from four electrodes). They reported no differences between dyslexic and control adults at 4 Hz or at 80 Hz, but a significant dyslexic deficit at 20 Hz. Poelmans et al. (2012) concluded that cortical processing of “phonemic-rate” auditory modulations was selectively impaired for adults with dyslexia.

As a test of TS theory, Hämäläinen et al. (2012) played AM white noise at four rates (2, 4, 10, and 20 Hz) to adults with and without dyslexia. They reported significantly reduced phase entrainment for the dyslexic participants in right hemisphere networks, but for the 2-Hz rate only. They also found significantly weaker right hemisphere entrainment overall (summed across modulation rates) for the dyslexic participants, and significantly stronger entrainment to the 10-Hz rate in the left hemisphere, a finding that was not predicted. This could indicate compensatory entrainment at faster temporal rates in those with dyslexia, consistent with the finding reported (bilaterally) by Lehongre et al. (2011) for rates above 40 Hz.

One possible reason for the discrepant findings across these studies using nonspeech stimuli is that participants had learned to read different languages, French (Lehongre et al., 2011), Dutch (Poelmans et al., 2012), and English (Hämäläinen et al., 2012; McAnally & Stein, 1997; Menell et al., 1999). However, this body of work has also been critiqued for the reliance on non-speech stimuli: the atypical patterns of entrainment may lack linguistic relevance. For the EEG paradigms used by McAnally and Stein (1997), Menell et al. (1999), and Poelmans et al. (2012), low spatial resolution is a further issue (due to the sparse electrode montage). Accordingly, it is important to examine neural entrainment to rhythmic speech rather than non-speech stimuli, which may have greater ecological validity and clinical relevance. To address this gap in the adult literature, here we utilised a rhythmic syllable repetition task previously used with dyslexic children (Keshavarzi et al., 2022; Power et al., 2013) and neurotypical infants (Ni Choisdealbha et al., 2023). The current study investigated responses in the beta and low gamma bands in addition to the delta and theta bands, to investigate whether atypical entrainment for dyslexic adults would be found at 20 Hz (Poelmans et al., 2012) or near 35 Hz (Lehongre et al., 2011) for a rhythmic speech input.

Previous studies have also reported abnormal neural oscillatory band power for children with dyslexia (Arns et al., 2007; Cutini et al., 2016; Keshavarzi, Mandke, et al., 2024; Power et al., 2016). Band-power analyses quantify neural oscillation power within distinct frequency ranges, providing insights into the strength of neural responses. Another common neural measure in speech processing studies is cross-frequency coupling, encompassing PAC and PPC (Giraud & Poeppel, 2012; Gross et al., 2013). PAC and PPC have not to our knowledge previously been explored in studies of adult dyslexia. PAC refers to the interaction where the phase of a lower-frequency oscillation is related to the amplitude of a higher-frequency oscillation (Tort et al., 2008). PAC may facilitate communication between neural networks operating in different frequency bands (Canolty & Knight, 2010). Abnormalities in PAC, particularly theta–gamma PAC, are believed to disrupt the timing and coordination of the neural activity necessary for phonological processing and reading fluency (Archer et al., 2020). In infant longitudinal studies, infants with stronger theta–gamma PAC at age 4 months showed better language outcomes at age 2 years (Attaheri et al., 2024). PAC is a currently neglected aspect of neural temporal dynamics in the adult dyslexia literature. PPC is a measure of the temporal synchrony between oscillations in different frequency bands (Lachaux et al., 1999; Tass et al., 1998) and is also lacking in current studies of adult dyslexia. In a speech context, differences in PPC could reflect impairments in the timing and coordination of neural processes that are important for efficient auditory and phonological processing.

Each oscillatory band is thought to serve a specific role in the neural processing of speech. Delta and theta oscillations are involved in processing the rhythmic characteristics of speech and are particularly important for phrasal (delta) and syllabic (theta) processing (Giraud & Poeppel, 2012). Infant EEG research suggests that these low-frequency bands are the foundational components necessary for the development of an efficient language system (Attaheri et al., 2024; Keshavarzi, Choisdealbha, et al., 2024), as infants show significant entrainment to natural speech in the delta and theta bands, but not in the alpha band. In infant longitudinal EEG studies, individual differences from age 2 months in preferred phase in the rhythmic syllable repetition task were found to predict differences in vocabulary and phonology at age 2 years (Ni Choisdealbha et al., 2023). Beta oscillations are implicated in motor functions (Engel & Fries, 2010) and play a significant role in top–down attentional regulation and sensory event prediction (Arnal et al., 2015). In the music domain, beta-band oscillations have been suggested to play a key role in representing the temporal structure of regular sound sequences (Fujioka et al., 2012, 2015). Low-gamma oscillations are thought necessary for encoding the rapid acoustic information necessary for phonemic representation (Giraud & Poeppel, 2012; Lehongre et al., 2011). These fast oscillations may support encoding of the fine-grained temporal details essential for distinguishing rapid transitions in speech, such as formant transitions. Disruptions in gamma-band activity have been observed for individuals with dyslexia with nonspeech stimuli (Lehongre et al., 2011). Lehongre et al. (2011) interpreted atypical gamma band entrainment to AM noise as a neural explanation for dyslexic difficulties in phoneme-level processing.

Critically, studying adults with dyslexia provides an opportunity to examine the developmental trajectory of temporal sampling mechanisms. If atypical low-frequency entrainment reflects a core causal mechanism of dyslexia, then such difficulties should still be present in adulthood despite extensive reading experience. Alternatively, developmental compensation may lead to reorganisation of temporal processing, for example, shifting from delta-band to theta-band mechanisms, or altering the balance between slow and fast oscillatory dynamics. Therefore, investigation of adults with dyslexia can provide evidence relevant to whether temporal sampling deficits persist into adulthood and can also generate developmental hypotheses about possible compensation or reorganisation mechanisms for future longitudinal research.

The use of audiovisual speech stimuli is also theoretically motivated. Natural speech perception is inherently multisensory, and visual articulatory cues (e.g., lip movements) provide robust low-frequency temporal information that can enhance neural entrainment (Chandrasekaran et al., 2009; Park et al., 2016; Schroeder et al., 2008). Such cues are particularly relevant for delta- and theta-band processing, which are central to TS theory. Accordingly, rhythmic audiovisual speech provides a more ecologically valid and temporally structured input than rhythmic non-speech stimuli. The 2-Hz presentation rate was chosen because it falls within the slow temporal range that is central to TS accounts, has previously been implicated in adult dyslexia in studies using AM nonspeech stimuli, and matches the rate used in our earlier child dyslexia studies. Our prior child studies compared dyslexic entrainment with syllables with a 2-Hz presentation rate in three conditions: visual-only speech, auditory-only speech, and audiovisual speech. Group differences were present only for the auditory-only and audiovisual conditions (Power et al., 2012, 2013). Accordingly, here we utilised only AV speech, which more closely reflects natural speech perception. The use of the same rhythmic audiovisual paradigm as in our previous studies with children with dyslexia (Keshavarzi et al., 2022; Power et al., 2013) also enabled a developmental comparison, albeit cross-sectional in nature. This comparison allowed us to assess whether the atypical neural responses identified in child samples are maintained, reorganised, or compensated in adulthood when the same temporally structured speech input is used. Because only a single stimulation rate was tested here, the present findings should be interpreted as specific to rhythmic audiovisual speech presented at 2 Hz, rather than assumed to generalise across all temporal rates relevant to speech processing. The current paradigm permits precise measurement of neural phase entrainment and preferred phase rather than providing a direct measure of speech-envelope tracking. Importantly, all neural analyses were restricted to the entrainment period prior to the occurrence of any rhythmic violation (see later for details). Accordingly, the reported neural measures primarily reflect responses to temporally regular audiovisual speech input rather than neural responses to rhythmic irregularities.

In summary, in the current study, a rhythmic audiovisual task (Keshavarzi et al., 2022; Keshavarzi, Richards, et al., 2024; Power et al., 2012, 2013) was administered to 24 adults with dyslexia and 24 adults without dyslexia while concurrently recording EEG data. We examined phase entrainment and neural oscillatory band power within the delta (0.5–4 Hz), theta (4–8 Hz), beta (15–25 Hz), and low gamma (25–40 Hz) frequency bands. Furthermore, PPC and PAC were assessed between delta–theta, delta–beta, delta–gamma, theta–beta, and theta–gamma frequency pairs for each group independently. Consistent with our previous findings using the syllable repetition paradigm with dyslexic children (Keshavarzi, Mandke, et al., 2024), we hypothesised *a priori* that the dyslexic group would exhibit an absence of consistent neural phase entrainment in the beta and gamma frequency bands. Based on TS theory and previous findings from Power et al. (2013) and Keshavarzi et al. (2022), we predicted that the preferred entrainment phase in both delta and theta bands would differ significantly between dyslexic and control groups. We further predicted significantly elevated band power for the dyslexic group in the delta frequency band both across the entire brain and within the right temporal region (Arns et al., 2007; Cutini et al., 2016), as well as in the beta frequency band across the whole brain (Keshavarzi, Mandke, et al., 2024; Power et al., 2016). No significant group differences were anticipated for delta–beta PAC, as to date no differences have been found in studies of children with dyslexia (Keshavarzi, Mandke, et al., 2024; Power et al., 2016). We did not have specific hypotheses regarding group differences in PAC for delta–theta, theta–beta, delta–gamma, and theta–gamma bands, or in PPC for delta–theta, delta–beta, theta–beta, delta–gamma, and theta–gamma frequency pairs.

## 2 Methods and Materials

### 2.1 Participants

A total of 48 adult native English speakers were tested, comprising 24 participants diagnosed with dyslexia (mean age = 21.4, standard deviation (SD) ± 2.5 years, age range = 18.3–29.1 years) and 24 control participants without dyslexia (mean age = 21.9, SD = 2.6 years, age range = 18.7–29.2 years). Dyslexic participants were recruited via the Accessibility and Disability Resource Centre at the University of Cambridge. Dyslexic participants had previously received an official diagnosis of dyslexia and no new formal clinical diagnosis was made at the time of adult testing, but group membership was supported by performance on the Test of Word Reading Efficiency, Second Edition (TOWRE-2; Torgesen et al., 2012). The inclusion criteria required that dyslexic individuals had no comorbid learning disabilities, such as autism spectrum disorder, dyspraxia, attention-deficit/hyperactivity disorder, or developmental language disorder, as verified through participant self-report. Additionally, dyslexic participants were required to perform at least 1 standard deviation below the control group mean on at least one subtest of the TOWRE-2. Control participants were matched closely to the dyslexic group in terms of age and nonverbal IQ, measured using the Matrix Reasoning subtest (Wechsler, 2008; see Table 1). There were no significant group differences in age or Matrix Reasoning scores. All participants underwent audiometric screening, assessing pure-tone auditory sensitivity bilaterally at frequencies of 250, 500, 1000, 2000, 4000, and 8000 Hz. Participants were required to have auditory thresholds of 20 dB HL or better. All participants provided written informed consent prior to their involvement, consistent with ethical guidelines set forth by the Declaration of Helsinki. The study was reviewed by the Psychology Research Ethics Committee at the University of Cambridge and received a favourable opinion.

**Table 1.** Mean scores (standard deviations) for age and behavioural measures for the control and dyslexic groups.

	Control	Dyslexia	Wilcoxon rank-sum test
Age	21.9 (2.6)	21.4 (2.5)	*p* = 0.55
TOWRE-SWE	118 (12.9)	94 (9.7)	*p* = 1.1×10^-6^
TOWRE-PDE	119 (8.9)	98 (8.3)	*p* = 5.8×10^-8^
Matrix reasoning	15 (1.6)	15 (1.8)	*p* = 0.53
TAN	76 (10)	72 (11.6)	*p* = 0.22

Measures were age, single word reading accuracy and nonword decoding (TOWRE-SWE, TOWRE-PDE), nonverbal IQ (Matrix Reasoning), and mathematical ability (TAN). Group differences were assessed using two-tailed Wilcoxon rank-sum tests.

### 2.2 Experimental set-up and stimuli

**Fig. 1.**
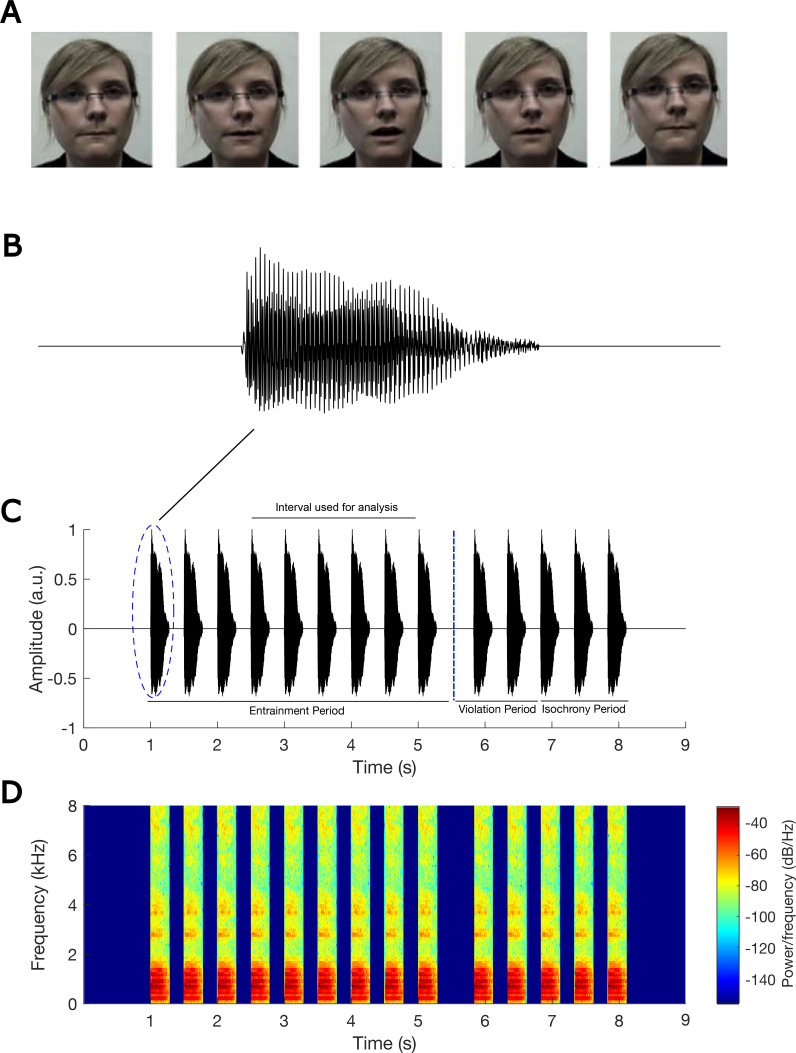
Experimental stimuli for the audiovisual task. Panel (A) shows a series of visual frames corresponding to a single articulation of the syllable “ba”. Panel (B) presents the acoustic waveform of one “ba” syllable. Panel (C) depicts a representative trial comprising a sequence of 14 “ba” stimuli, with a rhythmic deviation introduced at the 10th position. Irrespective of whether the rhythmic deviation was introduced at the 9th, 10^th^, or 11th position, the analysis window extended from the onset of the 3rd “ba” to 0.5 s after the onset of the 8th “ba” (a total duration of 2.5 s). Panel (D) illustrates the corresponding spectrogram for the auditory sequence shown in Panel (C). This figure is reproduced with permission from Keshavarzi et al. (2022).

The experimental design and stimulus presentation followed the procedures described in Keshavarzi et al. (2022) and Keshavarzi, Richards, et al. (2024). Following EEG cap placement, participants were seated in an electrically shielded, soundproof room. They heard and watched audiovisual stimuli via earphones and on a screen, respectively. EEG signals were recorded at a sampling rate of 1 kHz using a 128-channel EEG system (HydroCel Geodesic Sensor Net). Electrode impedances were visually monitored throughout acquisition using the montage display in the Geodesic EEG recording software and were maintained within the manufacturer-recommended range for the Geodesic EEG system. Stimuli consisted of a video display of a female talker producing rhythmic sequences of the syllable “ba” at a 2-Hz rate. Each sequence comprised 14 syllables. The face remained visible between syllables. For each syllable, the onset of the talker’s visible articulatory movement preceded the onset of the auditory “ba” by 68 ms, consistent with natural speech timing. The auditory “ba” had a duration of approximately 280 ms. One of the syllables in each sequence (either the 9th, 10th, or 11th) was temporally shifted to create a rhythmic violation. Participants were instructed to focus on the speaker’s lips while attending to the auditory stream and to press a key when they detected a syllable presented out of rhythm. The shift varied across trials. A 3-down 1-up adaptive staircase procedure was employed to determine the shift that was just noticeable for each participant. When a participant correctly identified the rhythmic violations on three consecutive trials, the deviation from the isochronous 500-ms stimulus-onset asynchrony (SOA) was reduced by 16.67 ms. The adaptive staircase started with an initial temporal deviation of 100 ms (six steps of 16.67 ms). A single failure to detect a violation led to an increase in the SOA of 16.67 ms. This procedure was designed to track an accuracy of 79.4% (Levitt, 1971), ensuring consistent task difficulty across participants. Each participant completed 90 trials, including 15 randomly interspersed catch trials containing no rhythmic deviation. Each trial was segmented into three distinct phases: an entrainment period, a violation period, and a return-to-isochrony period (see Fig. 1). The EEG recording session, excluding setup time, lasted approximately 15 minutes.

### 2.3 EEG data pre-processing and analyses

The EEG recordings were initially referenced to the Cz channel and subsequently subjected to band-pass filtering (0.2–42 Hz) using a zero-phase finite impulse response filter with 6-dB down points at 0.1 Hz and 47.25 Hz, as implemented through the MNE-Python package (Gramfort et al., 2013). Bad channels were detected manually from inspection of the spectrum and time–domain representations of the channel signals, followed by spatial interpolation (MNE-Python). Independent Component Analysis (ICA) employing the Infomax algorithm (MNE-Python) was performed on individual participant datasets. The resulting independent components were manually evaluated to identify and remove artefactual components, including eye blinks, horizontal and vertical eye movements, and heartbeat activity, based on inspection of their scalp topographies, power spectra, and time–domain waveforms. After preprocessing, the mean number of trials retained for analysis was 85.4 (SD = 2.2) for the control group and 84.5 (SD = 2.4) for the dyslexic group. Following preprocessing procedures, the EEG data were downsampled to 200 Hz and filtered into delta (0.5–4 Hz), theta (4–8 Hz), beta (15–25 Hz), and low gamma (25–40 Hz) bands (using MNE-Python). The data were then epoched into individual trials, from 0.5 s before the onset of the first “ba” stimulus to 4 s after. To reduce computational costs, the data were downsampled to 100 Hz. The analyses were restricted to EEG responses corresponding to the 4th–8th “ba” stimuli within a 2.5 s entrainment window (from the onset of the 3rd “ba” to 0.5 s after the onset of the 8th “ba”), ensuring that the rhythmicity had established.

### 2.4 Computation of phase entrainment

To evaluate phase entrainment for each group across frequency bands, the following procedure was applied, following Keshavarzi et al. (2022) and Keshavarzi, Richards, et al. (2024):

1. The instantaneous phase for each EEG channel was computed at the time points corresponding to the onsets of the five target “ba” stimuli (4th–8th) within each trial. The instantaneous phase φ(*t*) of a signal *s*(*t*) was calculated using the Hilbert transform, as follows:
  $$\varphi \left(t\right)=\arctan\left(\frac{s_{H}\left(t\right)}{s\left(t\right)}\right),$$ (1)
  where $s_{H}\left(t\right)$ is the Hilbert transform of signal *s*(*t*), defined by
  $$s_{H}\left(t\right)=\;\frac{1}{\pi }\int_{-\infty }^{\infty }\frac{s\left(\tau \right)}{t-\tau }d\tau .$$ (2)
2. For each trial, the mean phase was computed by averaging the instantaneous phase values across all EEG channels and the five “ba” stimuli using the circular mean (rather than an arithmetic mean), resulting in a single phase value per trial.
3. For each trial, a single unit vector (magnitude = 1) was placed in the vector space, with its angle determined by the phase value obtained in step 2.
4. The mean vector for each participant was then calculated by averaging the unit vectors from step 3 across trials, resulting in a single vector per participant, referred to as the *individual resultant vector*. The angle of the individual resultant vector is called the *individual preferred phase*, and its length serves as a measure of phase consistency strength across trials for each participant.
5. A single unit vector (magnitude = 1)—whose angle was determined by the angle of the *individual resultant vector*—was considered in the vector space for each participant.
6. Finally, the mean vector for each group was calculated by averaging across the unit vectors from step 5, resulting in a single vector called the *group resultant vector*. The angle of the *group resultant vector* is called the *group preferred phase*.

### 2.5 Computation of the band power

To investigate the band power of neural responses over the time interval used for analysis (See Fig. 1C), the following steps were conducted for each individual and each group:

1. The broadband power spectral density (PSD) was computed for each EEG channel using the *pspectrum*() function in MATLAB.
2. For each trial, the broadband PSD was calculated by averaging across the broadband PSDs obtained (in step 1) for all EEG channels.
3. For each participant, the broadband PSD was calculated by averaging across the broadband PSDs obtained (in step 2) for all trials of that participant.
4. The PSD for each frequency band (delta, theta, beta, and low gamma) and for each participant was calculated based on the broadband PSD obtained in step 3.
5. The PSD for each group and each frequency band (delta, theta, beta, and low gamma) was calculated by averaging across the PSDs obtained (in step 4) for all participants in a given group.

### 2.6 Computation of power spectral topographic map

To compute the power spectral topographic map for each frequency band of interest (delta, theta, beta, and low gamma), the following steps were performed:

1. To ensure data quality, outer EEG channels (E17, E48, E119, E125, E126, E127, E128) were excluded.
2. The PSD for each epoch was computed using Welch’s method across a broad frequency range (0.2–42 Hz).
3. Spectral data were averaged within each group (dyslexic or control), and mean spectral power was then calculated for the delta, theta, beta, and low gamma bands.
4. Statistical comparisons were conducted to assess power differences between groups, with a specific focus on the right temporal region (EEG channels: E100, E101, E102, E107, E108, E109, E113, E114, E115, E116, E120, E121). The focus on the right temporal region was determined a priori based on previous findings in dyslexia and our specific hypothesis (Arns et al., 2007; Cutini et al., 2016). Accordingly, statistical analyses were restricted to this region, rather than guided by visual inspection of the scalp topographies.

### 2.7 Computation of cross-frequency phase–amplitude coupling (PAC)

Cross-frequency PAC refers to the relationship between the amplitude of a high-frequency oscillation and the phase of a low-frequency oscillation. The PAC was quantified using the modulation index (*MI*), as proposed by Tort et al. (2008) and Hülsemann et al. (2019):

$$MI=\frac{KL\;\left(U,X\right)}{logB},\;$$ (3)

where *B* (= 18) represents the number of bins, *U* denotes the uniform distribution, *X* is the empirical distribution of the data, and KL (U,X) is the Kullback–Leibler divergence, computed as

KL (U,X)=logB−H(P). (4)

Here, H(.) represents the Shannon entropy, and *P* is the vector of normalised mean amplitudes per bin which is calculated as

$$P\left(j\right)=\;\frac{{\bar{a}}_{j}}{\sum_{k=1}^{N}{\bar{a}}_{k}},\;j=1,\;\ldots ,\;N,$$ (5)

where ${\bar{a}}_{j}$ denotes the average amplitude within the *j*th bin, and *k* is the index over all *N* bins. Note that *P* is a vector containing *N* elements. PAC was computed at the single-channel level and then averaged across all EEG channels over the whole head to obtain participant-level estimates for statistical analysis.

### 2.8 Computation of cross-frequency phase–phase coupling (PPC)

Cross-frequency PPC refers to the phase synchrony between oscillations in two different frequency bands. To quantify this synchrony, we used the phase locking value (PLV), as described by Lachaux et al. (1999), defined as

$$PLV=\;\frac{1}{T}\left|\sum_{t=1}^{T}e^{-j\Delta \varphi \left(t\right)}\right|,$$ (6)

where | . | denotes the absolute value operator, T represents the number of time samples, and Δφ(t) is the phase difference, computed as

$$\Delta \varphi (t)=\varphi_{1}(t)-\varphi_{2}(t).$$ (7)

Here, $\varphi_{1}\left(t\right)$ is the instantaneous phase of the first signal, and $\varphi_{2}\left(t\right)$ is the instantaneous phase of the second signal. PPC was computed at the single-channel level and subsequently averaged across all EEG channels to yield participant-level estimates for statistical analysis.

### 2.9 Statistical analyses

To assess phase-entrainment consistency within each group, Rayleigh tests of uniformity were applied to the individual preferred phases for each frequency band. Group differences in preferred phase were assessed using one-sample tests for the mean angle, following Keshavarzi et al. (2022). For each frequency band showing significant phase entrainment in both groups, the group preferred phase of one group (control) was used as the reference angle, and the individual preferred phases of the other group (dyslexia) were tested against this reference direction. The null hypothesis was that the mean direction of the tested group did not differ from the reference group preferred phase. Group differences in phase-entrainment consistency were assessed by comparing the lengths of the individual resultant vectors using two-sample *t*-tests, while within-group comparisons of delta- and theta-band consistency were conducted using paired-sample *t*-tests. For band-power analyses across the whole brain, group comparisons were performed using two-tailed Wilcoxon rank-sum tests. For topographic power analyses, inferential statistics were restricted a priori to the predefined right temporal region and were likewise assessed using two-tailed Wilcoxon rank-sum tests. Group differences in PAC and PPC were also evaluated using two-tailed Wilcoxon rank-sum tests for each frequency–band pairing. Because no PAC or PPC comparison was significant at the uncorrected level, multiple-comparison correction would not have altered the interpretation of these null findings. Behavioural thresholds in the rhythmic violation task were compared between groups using a two-sample *t*-test. Statistical significance was set at *p* < 0.05, and effect sizes were reported as Cohen’s *d* for independent-samples *t*-tests, Cohen’s *dz* for paired-samples *t*-tests, and rank-biserial correlation effect sizes (*r*) for Wilcoxon rank-sum tests.

## 3 Results

### 3.1 Phase entrainment consistency for each group

**Fig. 2.**
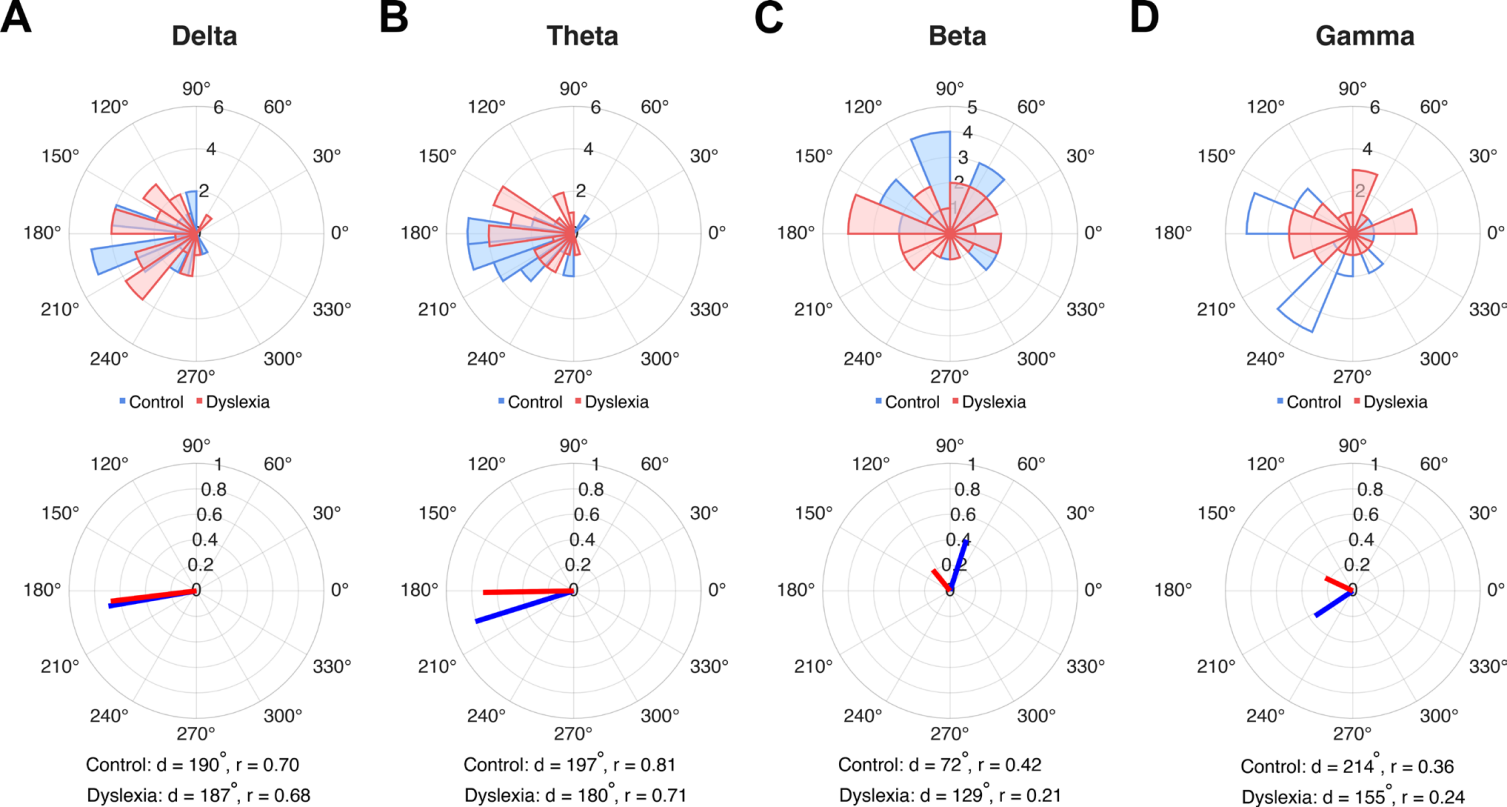
Preferred-phase distributions and group resultant vectors for the control and dyslexic groups across the delta (Panel A), theta (Panel B), beta (Panel C), and low-gamma (Panel D) bands. In the top row, light blue segments indicate the distribution of individual preferred phases for the control group and light red segments indicate the corresponding distribution for the dyslexic group. In the bottom row, blue and red lines indicate the group resultant vectors for the control and dyslexic groups, respectively. For each vector, d indicates the direction of the vector in degrees, corresponding to the group preferred phase, and r indicates the vector length, corresponding to the strength of phase consistency across participants within that group.

To assess the phase entrainment consistency within each group and across different frequency bands, we applied the Rayleigh test of uniformity to the *individual preferred phase*s (see Section 2.4) for each group and each band (see Fig. 2). For the control group, significant phase entrainment consistency was evident across all frequency bands: delta (*z* = 11.76, *p* = 1.6 × 10^-6^; see Fig. 2A), theta (*z* = 15.66, *p* = 5.4 × 10^-9^; see Fig. 2B), beta (*z* = 4.25, *p* = 0.013; see Fig. 2C), and low gamma (*z* = 3.04, *p* = 0.046; see Fig. 2D). For individuals with dyslexia, significant phase entrainment was found for the delta (*z* = 10.98, *p* = 4.53 × 10^-6^; see Fig. 2A) and theta (*z* = 12.16, *p* = 9.46 × 10^-7^; see Fig. 2B) bands. However, no consistent phase entrainment was observed for the beta (*z* = 1.09, *p* = 0.34; see Fig. 2C) and low gamma (*z* = 1.36, *p* = 0.26; see Fig. 2D) bands.

### 3.2 Comparison of preferred phase across groups

According to TS theory and to findings from Power et al. (2013) and Keshavarzi et al. (2022), we had hypothesised a group difference in preferred phase within the delta and theta bands. To compare group preferred phase between the control and dyslexic groups, we conducted separate one-sample mean direction tests following Keshavarzi et al. (2022), focusing only on frequency bands that showed significant phase entrainment. A significant difference in preferred phase was observed between groups for the theta band (*p* = 0.04), but not for the delta band (*p* = 0.77), contrary to our hypothesis.

### 3.3 Comparing the consistency of phase entrainment across groups

**Fig. 3.**
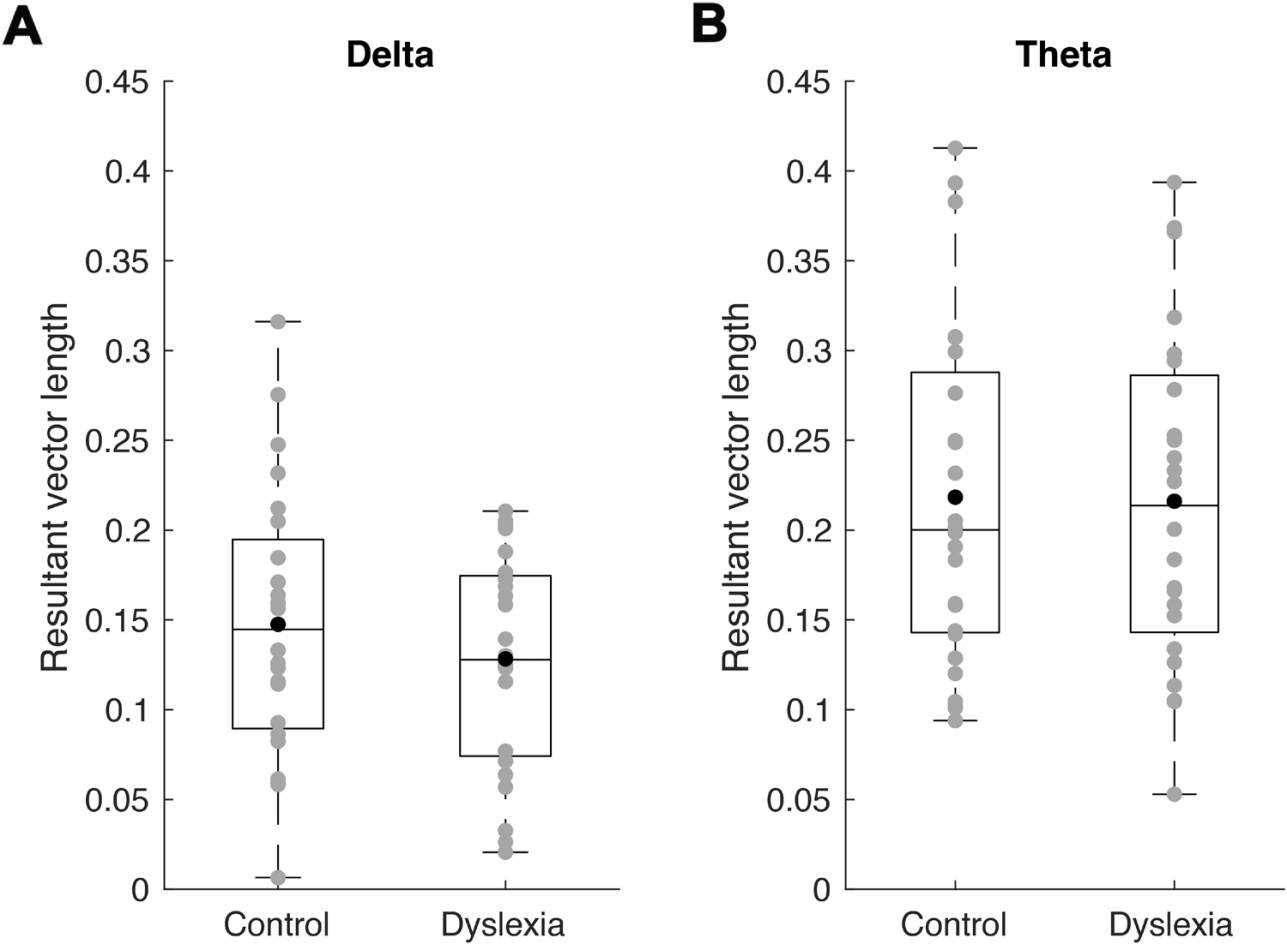
Boxplots illustrating the lengths of *individual resultant vectors*, representing the consistency of phase entrainment, for the delta (Panel A) and theta (Panel B) frequency bands. Grey circles show individual participant values, black circles indicate group means, and the horizontal line within each box marks the median. No significant group differences in phase entrainment consistency were observed for either the delta (Panel A) or theta (Panel B) bands. However, within both groups, theta-band entrainment exhibited significantly greater consistency than delta-band entrainment.

The length of each participant’s *individual resultant vector* (see step 4 in Section 2.4) reflects the consistency of phase entrainment across trials, longer vectors indicating higher within-subject consistency (Keshavarzi et al., 2022). To assess whether phase-entrainment consistency differed between groups, separate two-sample *t*-tests were performed on the lengths of the *individual resultant vectors*, limited to frequency bands that showed significant phase entrainment (see Fig. 3). No significant differences were found between the control and dyslexic groups for either the delta band (*p* = 0.33; Cohen’s *d* = 0.28; Fig. 3A) or the theta band (*p* = 0.93; Cohen’s *d* = 0.024; Fig. 3B). Phase entrainment consistency between the delta and theta bands was compared within each group. Theta-band entrainment showed significantly higher consistency than delta-band entrainment for both the control group (*p* = 0.006; Cohen’s *dz* = 0.80) and the dyslexic group (*p* = 3.1 × 10^−5^, Cohen’s *dz* = 1.09).

### 3.4 Comparing band power for the groups across the whole brain

**Fig. 4.**
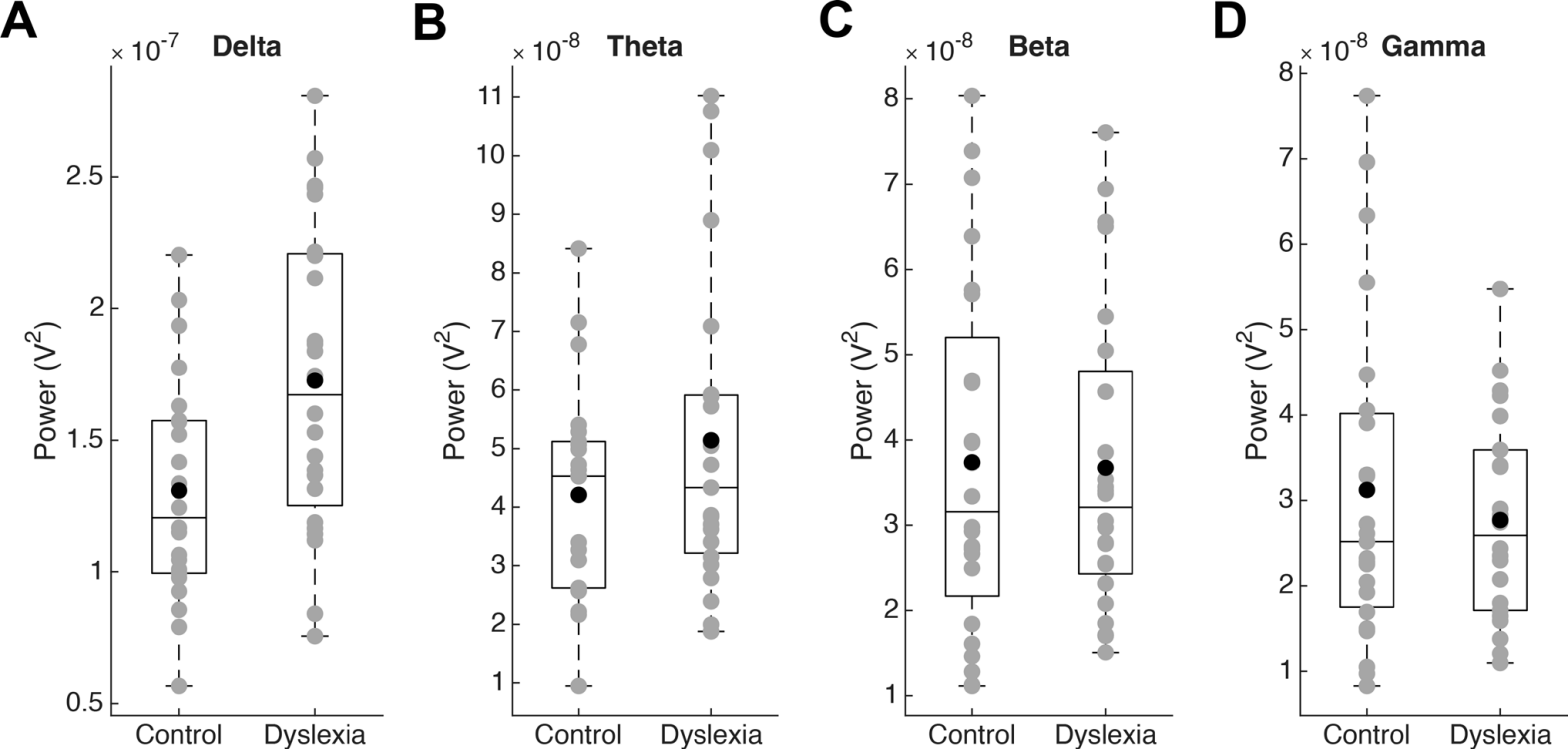
Boxplots of power values for the delta (Panel A), theta (Panel B), beta (Panel C), and low-gamma (Panel D) frequency bands over the analysis time window. Grey circles represent individual participants, black circles indicate group means, and the horizontal line within each box marks the median. Outliers were excluded from these plots. Note that the y-axis scales differ across panels to reflect the numerical range of power values in each frequency band.

The average power within the analysis time window (see Fig. 1) was calculated for each frequency band and group, following the method outlined in Section 2.5. Figure 4 presents boxplots of band-power values for the delta (Fig. 4A), theta (Fig. 4B), beta (Fig. 4C), and low gamma (Fig. 4D) bands, separately for the control and dyslexic groups. Based on our hypothesis, we expected group differences in delta- and beta-band powers. To test this, we applied two-tailed Wilcoxon rank-sum tests for each band, after excluding outliers. Outliers were defined as power values exceeding 1.5 times the interquartile range above the upper quartile or below the lower quartile within each group. The dyslexic group showed significantly higher power in the delta band than the control group (*z* = –2.43, *p* = 0.015; effect size: *r* = 0.36; 2 outliers in the control group), but no significant differences were found for the theta (*z* = –0.94, *p* = 0.35; effect size: *r* = 0.14; 2 outliers in the control group, 1 in the dyslexic group), beta (*z* = –0.09, *p* = 0.93; effect size: *r* = 0.01), and low-gamma (*z* = 0.01, *p* = 0.99; effect size: *r* = 0.00; 1 outlier in the control group, 2 outliers in the dyslexic group) bands.

### 3.5 Comparing spectral power topographic maps across groups

**Fig. 5.**
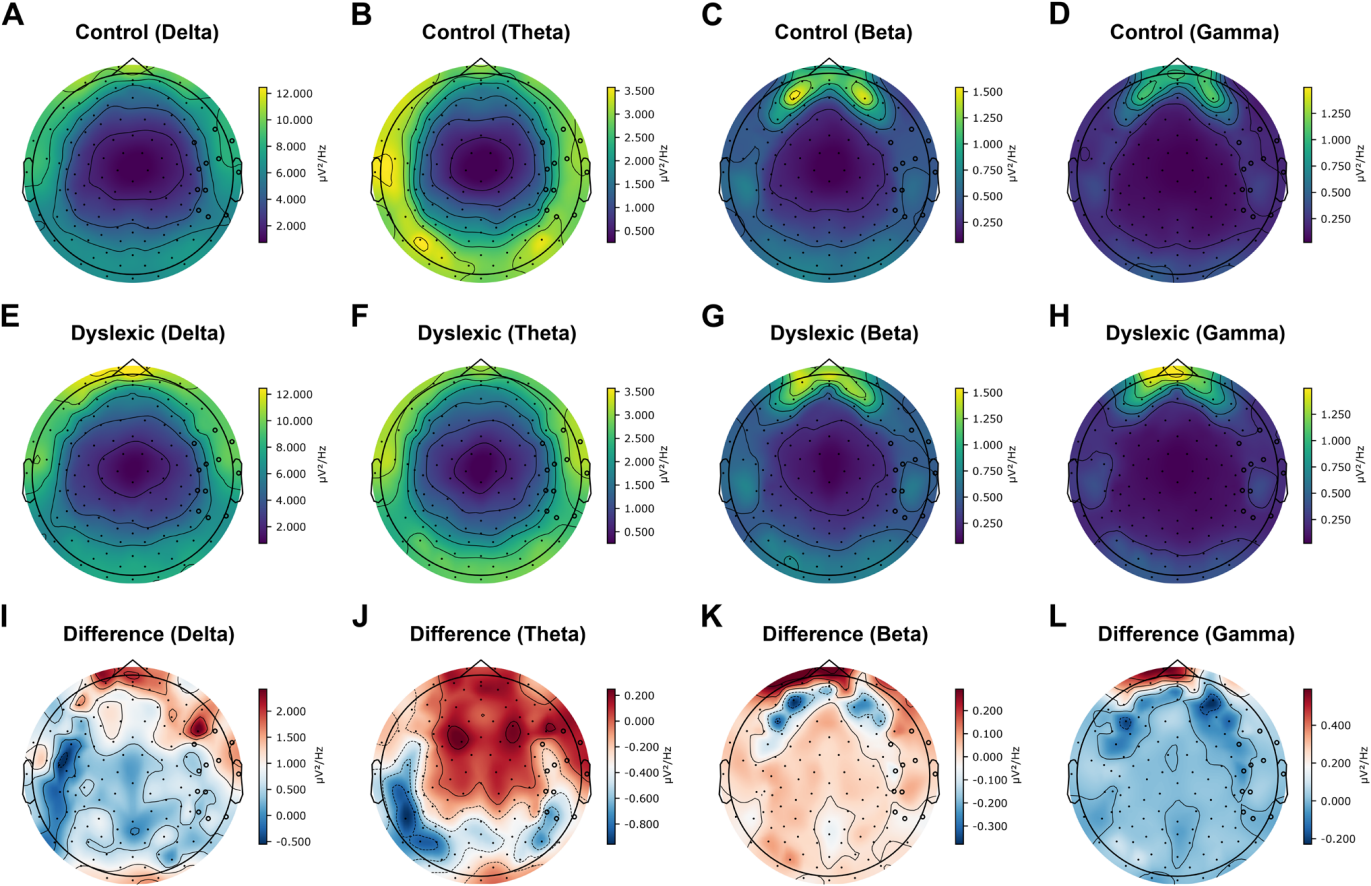
Descriptive visualisations of the spatial distribution of spectral power for the control and dyslexic groups, and the difference between them. Panels (A, B, C, D) display the delta, theta, beta, and low-gamma band power maps, respectively, for the control group. Panels (E, F, G, H) show the corresponding maps for the dyslexic group. Panels (I, J, K, L) present the difference maps (dyslexic minus control) for each frequency band. Open circles indicate the predefined right temporal electrodes used for the a priori regional statistical comparison. The control and dyslexic maps are shown using continuous sequential colour scales, whereas the difference maps are shown using a continuous diverging red-white-blue colour. Colour bars to the right indicate the range of spectral power values. Note that the colour scales vary across bands.

Topographic maps were calculated across the entire scalp for each frequency band and group. Figure 5 displays these maps for the delta, theta, beta, and low-gamma bands for the control group (Panels A–D), the dyslexic group (Panels E–H), and the difference between groups (Panels I–L). Based on previous findings by Cutini et al. (2016) and Arns et al. (2007), we hypothesised a group difference in delta-band PSD in the right temporal region. To test this, we conducted two-tailed Wilcoxon rank-sum tests for each frequency band within that region. A significant group difference was found for the delta band (*p* = 0.04; effect size: *r =* 0.30), but not for the theta (*p* = 0.48; effect size: *r =* 0.10), beta (*p* = 0.50; effect size: *r =* 0.10), or low-gamma (*p* = 0.32; effect size: *r =* 0.15) bands. For descriptive purposes only, Supplementary Figure S1 shows the distribution of electrodes exhibiting nominal channel-wise group differences across the whole scalp.

### 3.6 Comparison of cross-frequency phase-amplitude coupling (PAC) across groups

To examine group-level differences in cross-frequency PAC in response to rhythmic audiovisual stimuli, PAC values were calculated for five frequency-band pairings: delta–theta, delta–beta, theta–beta, delta–gamma, and theta–gamma (see Supplementary Fig. S2), using the procedure outlined in Section 2.7. These PAC measures were computed separately for the control and dyslexic groups. Group differences in cross-frequency PAC were statistically evaluated using two-tailed Wilcoxon rank-sum tests for each frequency pairing. There were no significant group differences for the delta–theta PAC (*z* = –1.0, *p* = 0.32; effect size: *r =* 0.14; Supplementary Fig. S2A), delta–beta PAC (*z* = –0.69, *p* = 0.49; effect size: *r =* 0.10; Supplementary Fig. S2B), theta–beta PAC (*z* = 0.03, *p* = 0.98; effect size: *r =* 0.00; Supplementary Fig. S2C), delta–gamma PAC (*z* = –0.69, *p* = 0.49; effect size: *r =* 0.10; Supplementary Fig. S2D), or the theta–gamma PAC (*z* = 0.13, *p* = 0.89; effect size: *r =* 0.02; Supplementary Fig. S2E). As no uncorrected comparison reached significance, the interpretation of the PAC results would be unchanged by multiple-comparison correction.

### 3.7 Comparing cross-frequency phase–phase coupling (PPC) across groups

PPC values were calculated for the following frequency band pairs: delta–theta, delta–beta, theta–beta, delta–gamma, and theta–gamma, separately for each group (see Supplementary Fig. S3), using the procedure outlined in Section 2.8. To compare PPC values between groups, two-tailed Wilcoxon rank-sum tests were performed for each band pair. No significant group differences were found for any PPC measure: delta–theta (*z* = –0.18, *p* = 0.86; effect size: *r =* 0.03; Supplementary Fig. S3A), delta–beta (*z* = –0.67, *p* = 0.50; effect size: *r =* 0.10; Supplementary Fig. S3B), theta–beta (*z* = –0.38, *p* = 0.70; effect size: *r =* 0.06; Supplementary Fig. S3C), delta–gamma (*z* = –0.59, *p* = 0.56; effect size: *r =* 0.09; Supplementary Fig. S3D), or theta–gamma (*z* = –0.09, *p* = 0.92; effect size: *r =* 0.01; Supplementary Fig. S3E). As no uncorrected comparison reached significance, the interpretation of the PPC results would be unchanged by multiple-comparison correction.

### 3.8 Comparing minimum detectable temporal delays in the audiovisual task (2 Hz) between the two groups

The mean temporal delay thresholds, determined using the adaptive staircase task (see Section 2.2), in which rhythmic sequences of the syllable “ba” were presented with both auditory and visual cues, were 74.3 ms for the control group and 78.5 ms for the dyslexic group. A two-sample *t*-test showed no significant difference between the groups (*p* = 0.64).

## 4 Discussion

This study was designed to examine whether atypical temporal sampling in adults with dyslexia is confined to low-frequency oscillations (<10 Hz, as proposed by TS theory), extends to faster temporal rates (beta and low gamma; Lehongre et al., 2011; Poelmans et al., 2012), or reflects a broader alteration in neural processing across multiple timescales. We, therefore, examined several neural measures (Keshavarzi, 2025): phase entrainment to index temporal alignment to rhythmic input, band-power analyses to assess the magnitude of oscillatory engagement within each frequency band, and cross-frequency coupling measures to evaluate coordination across bands. The data revealed significant phase entrainment in the delta and theta bands for both the adult dyslexic and control groups, consistent with previous findings for children with dyslexia (Keshavarzi et al., 2022; Power et al., 2013). The expected group difference in preferred phase of the delta band, as previously reported in studies of children (Keshavarzi et al., 2022; Power et al., 2013), was not found. Instead, the data revealed a significant group difference in the preferred phase in the theta band. As expected from previous studies using the rhythmic syllable repetition task with children, significant phase entrainment in the beta and gamma bands was absent for the adults with dyslexia. These data are supportive of both low- and high-frequency temporal sampling differences in adults with dyslexia, which are next discussed in more detail.

The group difference in preferred phase in the theta band is consistent with TS theory, which predicts atypical processing of speech-related information <10 Hz. Here it may reflect a temporal delay in neural processing for the individuals with dyslexia. Rather than indicating a simple shift from atypical delta-band to atypical theta-band processing with development, the difference in preferred phase may suggest that the neural mechanisms supporting rhythmic speech processing differ between children and adults. This could arise as a function of many factors including maturation, language experience, and literacy. For individuals with dyslexia, this phase difference in theta-band activity may reflect impairments in the precise temporal processing of speech units made more salient by learning to read. It is also possible that developmental differences in reading and language experience shape how low-frequency neural oscillations are engaged during speech processing in adulthood. For typically developing children, increased theta inter-trial coherence in the rhythmic syllable repetition task is related to higher reading age and to higher standard scores in word and nonword reading (Power et al., 2012). Reading experience over developmental time is necessarily reduced in adults with dyslexia, who also have lower standard scores than control participants in word and nonword reading.

Accordingly, the present adult findings may reflect changes in rhythmic processing strategies and neural entrainment profiles over development, rather than a direct developmental transition from delta-band to theta-band group differences. Longitudinal studies are required to investigate this possibility further. The literate adult brain, with its highly developed orthographic/phonological connections, may rely more on neural mechanisms governed by the theta band during oral speech processing than relatively less literate adult brains, such as dyslexic brains. The atypical preferred phase of the dyslexic group observed here for the theta band during rhythmic speech processing is consistent with findings from Leong and Goswami (2014), who reported that rhythmic synchronisation at slow rates (~5 Hz; theta band) was atypical for adults with dyslexia during a behavioural tapping-to-nursery-rhymes task.

Consistent with a maturational account, theta-band phase entrainment consistency was significantly greater than that of delta-band consistency for both the dyslexic and control groups. This contrasts with findings for children, for whom no significant difference in phase entrainment consistency has been observed between the delta and theta bands, for either group (Keshavarzi et al., 2022). These results suggest that adults—regardless of dyslexic status—may rely more heavily on theta-band neural dynamics than on delta-band activity during speech processing, at least when processing rhythmic audiovisual stimuli composed of a single syllable. Auditory neuroimaging studies of adults have shown that neural activity in the theta frequency band closely tracks syllable onsets (Ding & Simon, 2014; Di Liberto et al., 2015; Keshavarzi et al., 2020). This contrast between adult and child neural data highlights the importance of considering developmental differences when interpreting neural entrainment in different frequency bands.

Significant phase entrainment was not observed in the beta band for the dyslexic adults, whereas the control group exhibited significant entrainment for this faster band. This finding is consistent with our previous results for dyslexic children (Keshavarzi, Mandke, et al., 2024). The absence of consistent beta-band phase entrainment for adults with dyslexia may indicate a reduced ability to track the timing of temporally regular events. This may reflect deficits in top–down regulatory mechanisms and in temporal prediction—functions typically associated with beta oscillations (Arnal et al., 2015). Indeed, Arnal et al. (2015) demonstrated that beta-band activity is critical for temporal prediction during speech processing in the adult brain, facilitating the alignment of neural oscillations with the timing of upcoming sensory targets, possibly via motor planning (Morillon et al., 2019). Given that Poelmans et al. (2012) also found reduced entrainment for dyslexic adults in the beta band in response to non-speech rhythmic stimulation (AM noise), the absence of consistent beta-band phase entrainment to rhythmic speech stimuli in dyslexia may reflect a difficulty with temporal prediction and sensorimotor integration, which affects the processing of both speech and non-speech inputs.

Consistent phase entrainment in the low-gamma band was observed only for the control group. Gamma oscillations have been argued to facilitate the encoding of phonemic elements within speech (Giraud & Poeppel, 2012). Coupled with the data reported by Lehongre et al. (2011) for French adults with dyslexia and AM noise, the EEG data presented here may indicate that gamma band deficits reflect broader auditory processing difficulties. A subsequent study by Lehongre et al. (2013) using natural speech reported that adult dyslexic readers showed reduced gamma responses in the left hemisphere during passive viewing of an audiovisual movie. These findings were replicated and extended by Lizarazu et al. (2021), who used MEG to demonstrate that dyslexic adults exhibited significantly reduced neural phase locking at 30 Hz—within the low-gamma range—to both speech and non-speech AM stimuli. Accordingly, the possibility of “phonemic oversampling”, which refers to an over-reliance on phoneme-rate temporal information, in adults with dyslexia remains open. It may also be that atypical low-frequency temporal sampling necessarily affects higher frequencies, given the mechanistic hierarchical nesting between slow (delta, theta) and fast (beta, gamma) oscillatory rates in auditory cortex during speech processing (Arnal et al., 2015; Gross et al., 2013).

Band-power analysis revealed significant differences between dyslexic and control participants for the delta band, both across the whole brain and in the right temporal region. Differences in the right temporal region may reflect generalised impairments in rhythmic processing, as this region is thought to preferentially process larger units, as reflected in speech and musical prosody (Boemio et al., 2005; Sammler et al., 2015). Both speech and non-speech neural rhythmic studies with children have shown differences in right temporal regions (Arns et al., 2007; Cutini et al., 2016). The absence of significant group differences in the theta and beta bands suggests that the amplitude of oscillations in these frequency ranges may be less affected than the amplitude of oscillations in the delta band for adults with dyslexia, at least in the context of rhythmic audiovisual stimulation. Importantly, the band-power analyses complement the phase-entrainment findings by showing that group differences were not limited to temporal alignment alone, but also involved the strength of oscillatory engagement, particularly in the delta band.

Our investigation of cross-frequency PPC and PAC revealed no significant differences between the dyslexic and control groups in delta–theta, delta–beta, theta–beta, delta–gamma, or theta–gamma interactions. These results suggest that synchrony between different neural oscillatory activities—as measured by PPC and PAC—is not significantly disrupted in adults with dyslexia, at least in response to rhythmic audiovisual stimuli. This finding may indicate that neural deficits in dyslexia are more closely related to the neural entrainment and processing of information within individual frequency bands than to cross-frequency interactions. However, it is also the case that coupling-strength measures used here for assessing PAC and PPC do not directly characterise phase direction or temporal polarity (see Keshavarzi, 2026). Specifically, neither MI-based PAC nor PLV-based PPC strength can distinguish phase relationships that differ by 180°. Accordingly, equivalent PAC or PPC strength values may not necessarily imply identical temporal organisation.

Importantly, neural differences were observed between groups despite the absence of significant group differences in behavioural thresholds in the rhythmic violation task. This suggests that adults with dyslexia may achieve comparable performance through compensatory or alternative processing strategies, even when the underlying neural dynamics of temporal alignment differ from those for control adults. Such a difference was observed in a prior adult study administering rhythmic tones at 2 Hz to participants with and without dyslexia, in which a button press was required whenever a tone was replaced by white noise (Soltész et al., 2013). In this study, control adults showed faster reaction times during the rising phase of the 2-Hz oscillation, whereas dyslexic adults showed no relationship between reaction time and oscillatory phase, despite the groups being matched for behavioural performance. The dissociation observed here between neural and behavioural measures is compatible with the developmental rationale of the study: temporal sampling differences may persist into adulthood, but their behavioural consequences may be reduced, masked, or compensated by maturation and experience.

A limitation of the present study is that only audiovisual speech stimuli were presented. Accordingly, although the paradigm captures neural responses to temporally structured multisensory speech, it does not permit the dissociation of auditory, visual, and audiovisual integrative contributions to the observed neural effects. Future studies with adults that include auditory-only and visual-only control conditions would be required to determine the extent to which the reported group differences reflect auditory temporal sampling, visual speech processing, audiovisual integration, or a combination of these processes.

Taken together, the present findings suggest that temporal sampling differences in dyslexia are neither confined to a single oscillatory band nor uniformly distributed across all frequency bands and neural measures. Instead, the pattern appears selective and multi-level. The findings most directly relevant to TS theory were the group differences in low-frequency entrainment, particularly the altered theta-band preferred phase. At the same time, atypical entrainment was evident at faster rates in the beta and low-gamma bands, and the band-power analysis revealed elevated delta-band activity for the dyslexic group. In contrast, cross-frequency PAC and PPC did not differ significantly between groups. This overall pattern is, therefore, consistent with a multi-timescale account of atypical neural dynamics in dyslexia in adulthood. It also suggests that the additional measures used here were valuable not because they all showed abnormalities, but because they helped to distinguish which aspects of neural temporal organisation were atypical and which were not.

In conclusion, this study investigated whether atypical temporal sampling in dyslexia in a rhythmic syllable repetition paradigm is confined to low-frequency oscillations, extends to faster oscillations, or reflects a broader alteration in neural processing across multiple timescales in adulthood. The rhythmic 2-Hz audiovisual paradigm selected here has been previously utilised with infants and children, for whom individual differences in preferred phase either predicted later language outcomes (Ni Choisdealbha et al., 2023), or showed atypicality in participants with dyslexia (Keshavarzi et al., 2022; Power et al., 2013). Here we observed significant phase entrainment in the delta and theta bands for both dyslexic and control adults, with a significantly different theta-band preferred phase for dyslexic individuals. This is consistent with atypical low-frequency speech processing in dyslexia as proposed by TS theory. Consistent with prior findings with dyslexic children (Keshavarzi, Mandke, et al., 2024), dyslexic adults showed an absence of consistent beta- and gamma-band phase entrainment. At the same time, no group differences in PAC and PPC were found. This suggests that the adult dyslexic profile is characterised by both low-frequency and high-frequency temporal sampling deficits. Further, both groups of adults exhibited greater theta-band phase entrainment consistency than delta-band entrainment, in contrast to findings with children. This is suggestive of developmental differences that may be linked to either maturation, increased language experience, increased reading experience, or all of these factors. The pattern observed is consistent with the possibility of developmental reorganisation, although longitudinal studies will be required to directly test this interpretation. Finally, significant group differences in delta-band power in the right temporal region may indicate atypical neural oscillatory responses to larger phonological units in speech (such as syllables) in dyslexic adults, consistent with the asymmetric sampling in time proposals of Boemio et al. (2005). Overall, the data suggest that atypical temporal sampling is present in adult dyslexia across multiple neural timescales.

## Supplementary Material

Supplementary Material

## Data Availability

De-identified participant data and analysis code are available from the corresponding author upon reasonable request, subject to institutional and ethical constraints.
