# Supplementary material for: Atypical low-frequency and high-frequency neural entrainment to rhythmic audiovisual speech in adults with dyslexia
Source: Imaging Neurosci (Camb). 2026 Aug 5;4:IMAG.a.1329. doi: 10.1162/IMAG.a.1329 (PMC13449930; doi:10.1162/IMAG.a.1329)
Supplement: Supplementary file 1 [file IMAG.a.1329_supp.pdf]

Supplementary Information

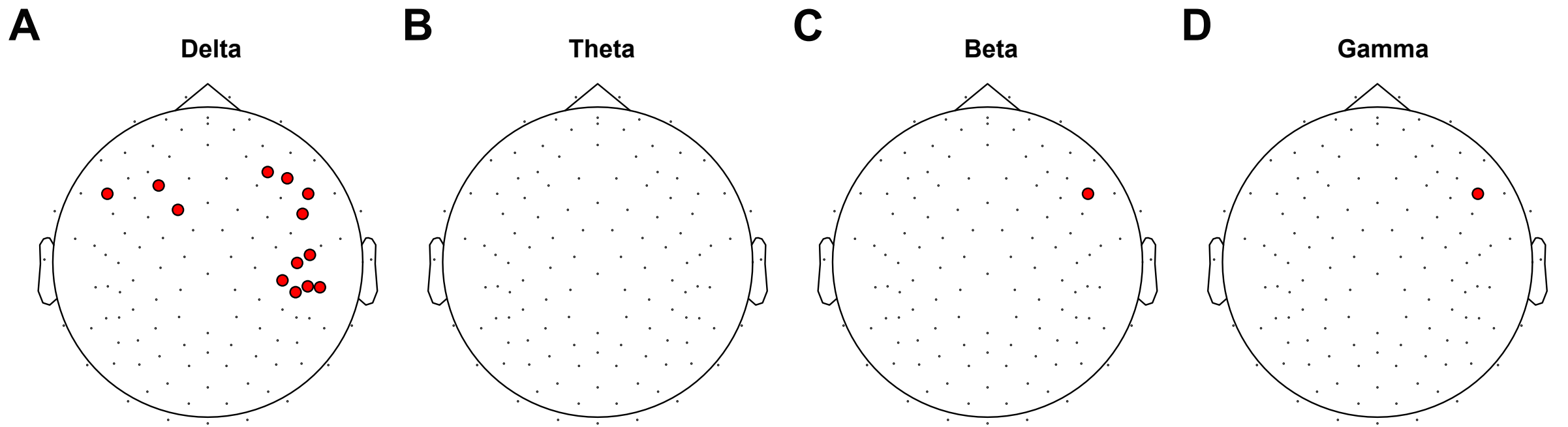

Figure S1. Electrodes showing nominal channel-wise group differences in spectral power for each frequency band. Red circles indicate electrodes with nominal two-sided Wilcoxon rank-sum test results of $p < 0.05$ (uncorrected), shown for descriptive purposes only. The formal statistical analysis reported in the manuscript was restricted a priori to the predefined right temporal region.

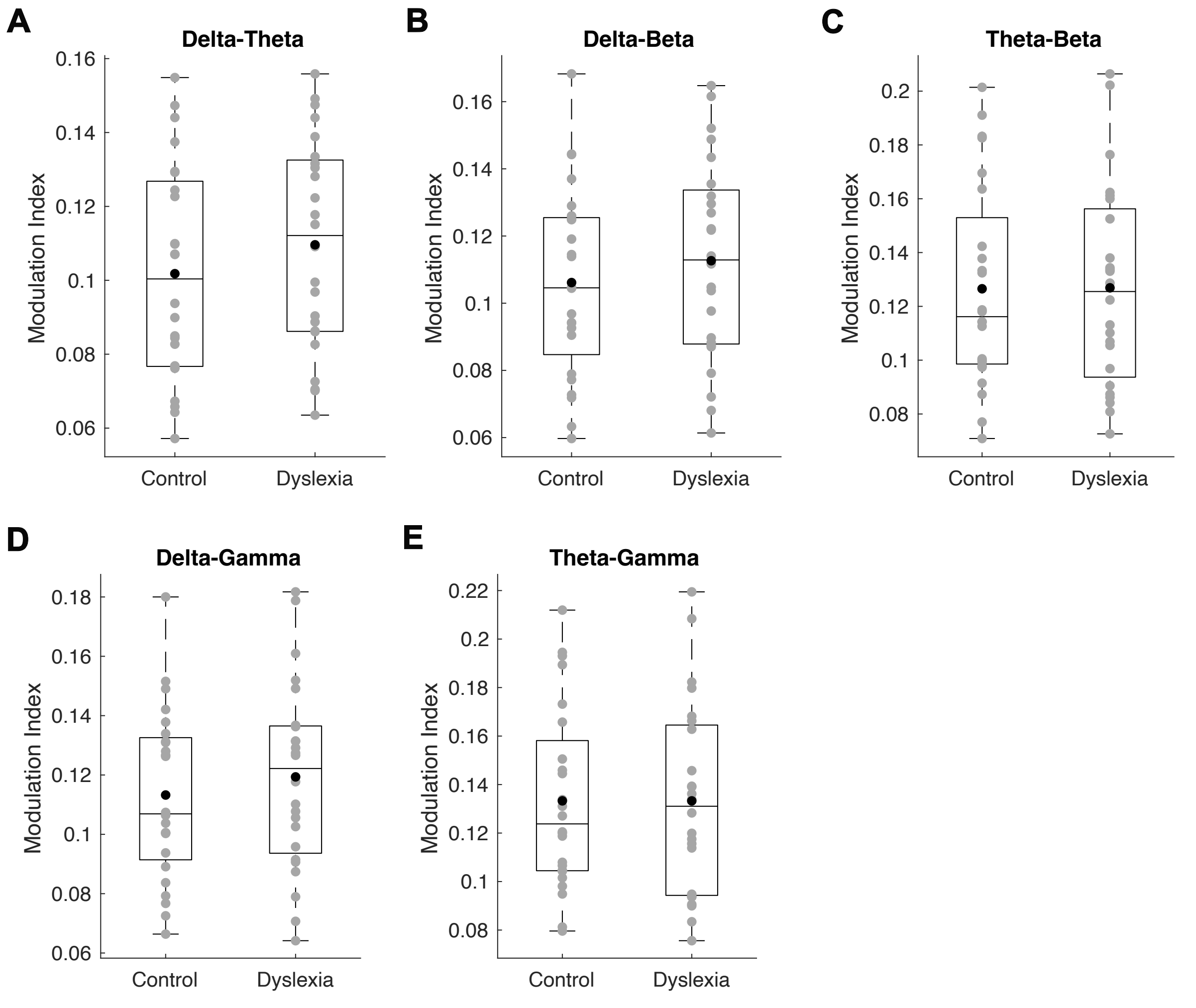

Figure S2. Boxplots of PAC values for each frequency band pair over the analysis time window: delta-theta (Panel A), delta-beta (Panel B), theta-beta (Panel C), delta-gamma

(Panel D), and theta-gamma (Panel E). Grey circles represent individual participants, black circles indicate the group means, and the horizontal line within each box denotes the median.

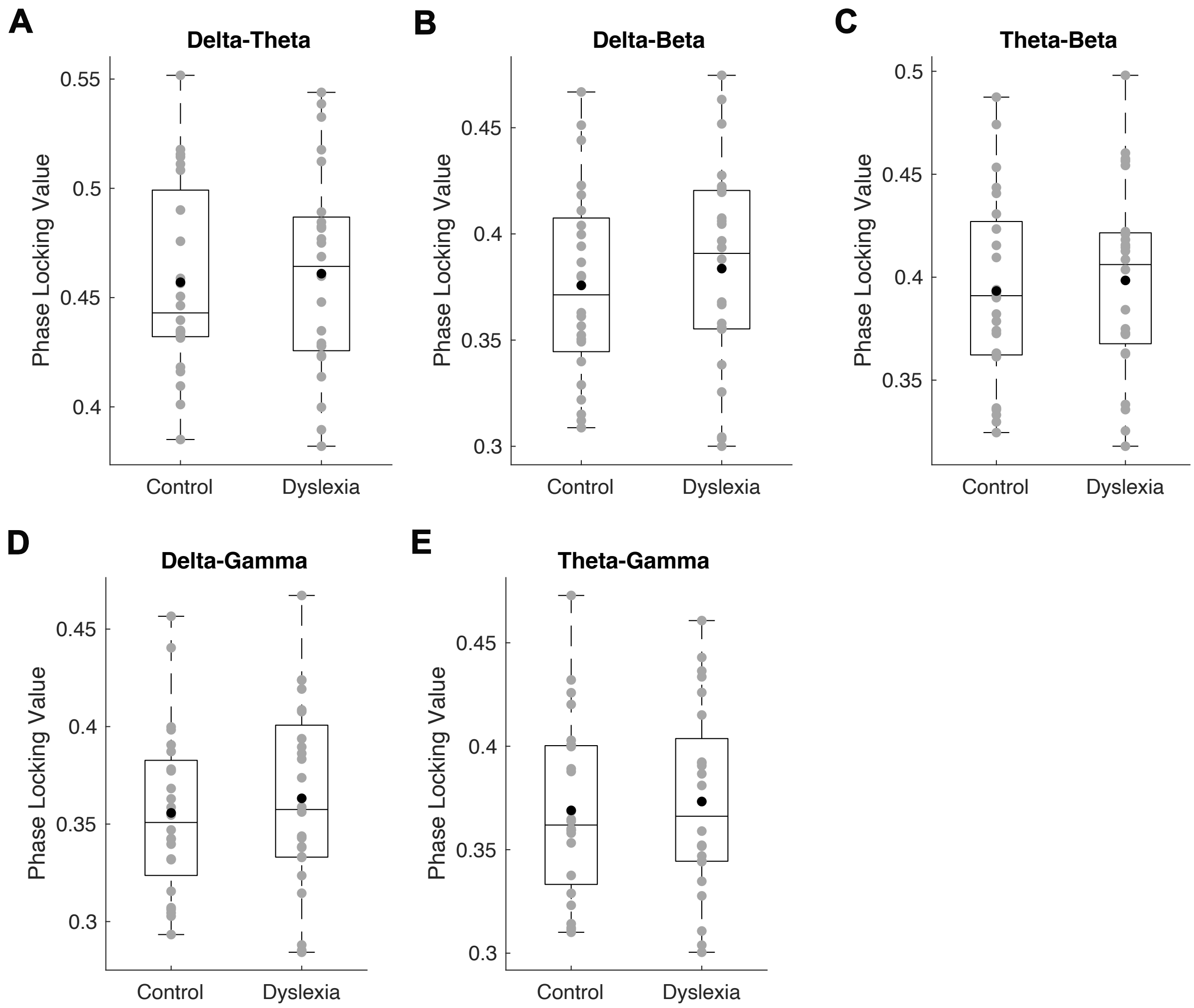

Figure S3. Boxplots of PPC values over the analysis time window for the following frequency band pairs: delta-theta (Panel A), delta-beta (Panel B), theta-beta (Panel C), delta-gamma (Panel D), and theta-gamma (Panel E). Grey circles represent individual participant values, black circles indicate group means, and the horizontal line within each box marks the median.
